# Untying chronic pain: prevalence and societal burden of chronic pain stages in the general population - a cross-sectional survey

**DOI:** 10.1186/1471-2458-14-352

**Published:** 2014-04-13

**Authors:** Winfried Häuser, Frederik Wolfe, Peter Henningsen, Gabriele Schmutzer, Elmar Brähler, Andreas Hinz

**Affiliations:** 1 Department of Internal Medicine I, Klinikum Saarbrücken, D-66119 Saarbrücken, Germany; 2 Department of Psychosomatic Medicine, Technische Universität München, D- 81675 München, Germany; 3 National Data Bank for Rheumatic Diseases and University of Kansas School of Medicine, Wichita, KS, USA; 4 Department of Medical Psychology and Medical Sociology, Universität Leipzig, Ph.-Rosenthal-Str. 55, D-04103 Leipzig, Germany; 5 Clinic for Psychosomatic Medicine and Psychotherapy, University Medical Center of the Johannes Gutenberg University, D-55131 Mainz, Germany

**Keywords:** Chronic pain, Disease load, Social inequality, General population, Cross-sectional survey

## Abstract

**Background:**

Chronic pain is a major public health problem. The impact of stages of chronic pain adjusted for disease load on societal burden has not been assessed in population surveys.

**Methods:**

A cross-sectional survey with 4360 people aged ≥ 14 years representative of the German population was conducted. Measures obtained included demographic variables, presence of chronic pain (based on the definition of the International Association for the Study of Pain), chronic pain stages (by chronic pain grade questionnaire), disease load (by self-reported comorbidity questionnaire) and societal burden (by self-reported number of doctor visits, nights spent in hospital and days of sick leave/disability in the previous 12 months, and by current unemployment). Associations between chronic pain stages with societal burden, adjusted for demographic variables and disease load, were tested by Poisson and logistic regression analyses.

**Results:**

2508 responses were received. 19.4% (95% CI 16.8% to 22.0%) of participants met the criteria of chronic non-disabling non-malignant pain. 7.4% (95% CI 5.0% to 9.9%) met criteria for chronic disabling non-malignant pain. Compared with no chronic pain, the rate ratio (RR) of days with sick leave/disability was 1.6 for non-disabling pain and 6.4 for disabling pain. After adjusting for age and disease load, the RRs increased to 1.8 and 6.8. The RR of doctor visits was 2.5 for non-disabling pain and 4.5 for disabling pain if compared with no chronic pain. After adjusting for age and disease load, the RR fell to 1.7 and 2.6. The RR of days in hospital was 2.7 for non-disabling pain and 11.7 for disabling pain if compared with no chronic pain. After adjusting for age and disease load, the RR fell to 1.5 and 4.0. Unemployment was predicted by lower educational level (Odds Ratio OR 3.27 [95% CI 1.70-6.29]), disabling pain (OR 3.30 [95% CI 1.76-6.21]) and disease load (OR 1.70 [95% CI 1.41-2.05]).

**Conclusion:**

Chronic pain stages, but also disease load and societal inequalities contributed to societal burden. Pain measurements in epidemiology research of chronic pain should include chronic pain grades and disease load.

## Background

Neck pain, low back pain and migraine belong to the nine leading specific causes of years lived with disability in 1990 and 2010 [1]. The high prevalence of chronic pain and its negative societal burden provide justification for regarding chronic pain as a public health priority [2,3]. However, prevalence estimates of chronic pain in population studies range from 11% to 64% in [3,4] leaving uncertainty about the real extent of the “pain problem” [5]. The wide range of prevalence rates of chronic pain was explained by the use of different definitions of chronic pain (e.g. three versus six months duration) and wording of the questions on chronic pain in the studies [6].

In addition, problems with the classification of chronic pain exclusively based on duration have been discussed. Studies with primary care patients suggested that a distinction between acute and chronic pain based on 3-month or 6-month duration may be arbitrary [7,8]. Additional criteria than duration such as disability should be considered when classifying chronic pain [8]. Instruments to differentiate between non-disabling and disabling pain have been developed [9], but were not used by most surveys on chronic pain in the general population [2,4].

Akin to many other chronic non-communicable diseases across the globe, chronic pain is typically accompanied by somatic and mental comorbidities [3]. Therefore the burden of chronic pain in terms of health care use or unemployment could be confounded with the one of associated diseases. Thus, there is a need for further research in this area [10].

In 2003, a computer assisted telephone survey on the prevalence and impact of chronic pain was conducted in 15 European countries including Germany. However, the refusal rate was 62% in Germany. 302 Germans with chronic pain participated in the survey whose demographic data were not reported. Therefore the validity of the results of this survey for Germany is questionable [11]. The German Health Surveys conducted in 1998 and 2012 by the Robert Koch Institute did not assess chronic pain in adults [12,13]. The 2012 German survey of our study group did not use validated instruments to assess chronic pain [14]. In addition, the prevalence of chronic pain stages has not been studied in Germany until now. Due to these research gaps, the aims of our study were to assess

a. The prevalence of chronic pain stages

b. The impact of chronic pain stages on key indicators of societal burden (days of sick leave/disability, health care use, unemployment) with and without adjustment for demographic variables and disease load in a cross-sectional survey of a representative German population sample.

## Methods

### Design and participants

The study was part of the annual general population surveys conducted by the University of Leipzig in which political and religious attitudes as well as health topics were assessed. The participants were informed that the study has no specific focus.

### Study participants, selection criteria, selection methods and selection procedures

A representative sample of the German population was selected with the assistance of a demographic consulting company (USUMA, Berlin, Germany). The random selection was based on multistage sampling. First, 258 sample point regions were randomly drawn from the last political election register, covering rural and urban areas from all regions in Germany. The second stage was a random selection of households using the random route procedure (based on a starting address). The third stage was a random selection of household respondents with the Kish selection grid. The sample was aimed to be representative in terms of age, gender, and education of the German population. The inclusion criteria for the study were age at or above 14 and the ability to read and understand the German language.

All subjects were visited by a study assistant and informed about the investigation. Subjects were presented with self-rating questionnaires. The survey included several questionnaires on somatic and psychological symptoms (health survey) as well as questionnaires on eating behavior, political attitudes and religious topics. The assistant waited until the participants answered all questionnaires, and offered help if persons did not understand the meaning of questions.

Data collection took place between May and June 2013. A first attempt was made at 4,360 addresses, and 2508 (57.5%) persons participated fully. Reasons for non-participation included the following: three unsuccessful attempts to contact the household or selected household member (14.8%); the household or selected household member declined to participate (13.6%); or the household member was on a holiday break (0.9%). Furthermore, 0.4% of the participants were excluded because they were not able to follow the interview because of illness, as were 12.4% who refused to finish the interview. 0.4% of interviews could not be analysed because of missing data.

### Questionnaires

#### Chronic pain and persistent bodily pain

Individuals with chronic nonmalignant pain were identified by screening questions based on the International Association of Pain definition of chronic pain (“pain that persists beyond normal tissue healing time, which is assumed to be 3 months”) [15], as follows: 1. “Did you experience pain that occurred constantly or flared up frequently in the last three months?” [16]. 2. Participants who answered “yes” were asked if the pain was attributable to a malignant disease. If the answer was yes, the participants were excluded from questions on pain grading. 3. The bodily pain item (“How much bodily pain have you had during the last 4 weeks? (no, very mild, mild, moderate, severe, very severe”)) of the Short Form Health Survey German version (SF-8) [17] was presented to every participant with a positive response to question 1. Chronic non-malignant pain was defined by a positive response in question 1, a negative response in question 2 and the report of at least very mild pain in question 3.

#### Chronic pain stages

Chronic pain stages were assessed using the Chronic Pain Grade questionnaire (CPG). The questionnaire enquires about current pain and pain over the previous three months. This seven-item scale then classifies pain into four hierarchical grades based on severity and disability: Grade I, low disability—low pain intensity; Grade II, low disability—high pain intensity; Grade III, high disability—moderately limiting; and Grade IV, high disability—severely limiting [9]. This questionnaire has been validated for use as a self-completion measure in postal surveys in the general population [18]. We used the validated German version of the CPG [19]. For the purpose of some analyses, individuals were classified into those with non-disabling chronic pain (CPG 1 and 2) and those with disabling chronic pain (CPG 3 and 4) [9].

#### Demographic questionnaire and body mass index

Age, gender, family status, educational level, net family income per months, current body weight and height were assessed by a standardized questionnaire used in previous German health surveys [14].

#### Disease load

The generic self-administered comorbidity questionnaire (SCQ) is a frequently used and validated instrument in clinical and health services research to assess common diseases which might impact functioning. The SCQ asks about the presence, treatment and functional limitations of 12 common diseases and three additional non-specified medical problems. Three subscale scores (present disease, present disease with drug treatment, present disease with associated disability and drug treatment) (0–15 each) and a total score (0–45) can be calculated [20]. We used the German version SCQ-D [21]. Osteoarthritis was substituted by alcohol – or drug abuse in a German version of SCQ (Sangha and Offenbächer, personal communication). We excluded “back problems” from all regression analyses because we aimed to assess the relative impact of non-painful comorbidities compared to chronic pain stages on societal burden.

#### Health care utilization

Subjects were asked to rate if and how frequently they visited doctors (general practitioners, pain management specialists and other medical specialists) during the previous 12 months for any reason. The total number of doctor visits in the prior 12 months was calculated. Moreover, hospitalisation (number of nights staying in hospital) was assessed. All items on health care utilization were assessed according to the German National Health Interview and Examination Survey [22].

### Statistical analysis

Because < 5% of items were missing we did not use imputation methods [23]. Up to one missing item in the disability score of the CPG was substituted by the individual rounded mean. If more items were missing, the questionnaire was excluded from analysis. Missing values in the SCQ and Health care utilization questionnaire were coded as zero.

Statistical analyses were conducted with the SPSS 19.0 statistical package and STATA 13.1. Absolute values and percentages were used for descriptive statistics of categorical data and means with standard deviations for descriptive statistics of continuous data. Despite multiple comparisons, alpha-level was set to 0.05 because of the exploratory and hypothesis-generating design of the study.

The associations between pain grades and days of sick leave age/inability to work, number of nights spent in hospital, number of family physician and specialist visits within the previous 12 months with and without control for age and disease load were assessed by Poisson regression analyses. Results are expressed as rate ratios (RR) with 95% confidence intervals (CIs). The associations between demographic factors, chronic pain grades and comorbidity index with unemployment were examined using binary logistic regression. Results are expressed as odds ratios (OR) with 95% confidence intervals (CIs).

### Ethics

All participants were informed about the study procedures and signed an informed consent form. For children under the age of 18 years the parents or guardians also gave written informed consent. The study was approved by the institutional ethics review board of the University of Leipzig (Az 092-12-05032012).

## Results

### Sample characteristics

The study sample was approximately representative of the general German population in terms of age groups, sex ratio and educational level [24] (see Additional file 1).

Demographic details of the study sample are outlined in Table 1.

**Table 1:** Demographic and clinical characteristics of the total study sample (N = 2508)

**Variable**	**N (%)**
**Gender**	
Female	1334 (53.2)
**Age [years]**	
Mean (SD)	49.7 (17.3)
**Family status**	
Married/living together	1315 (52.4)
**Education**	
No school finished	67 (2.7)
In school	78 (3.1)
Primary or secondary school	1898 (75.7)
High school or higher	455 (18.1)
Missing	10 (0.4)
**Current professional status**	
Working fulltime	996 (39.7)
Working partial time	310 (12.4)
Without job	142 (5.7)
Pensioner	745 (29.7)
Homemaker	42 (1.7)
Schooling or university education	150 (6.0)
Other (e.g. maternity leave)	19 (0.8)
Missing	104 (4.1)
**Family net income per month (€)**	
< 750	114 ( 4.5)
750 -1250	403 (16.1)
1251-2500	735 (29.3)
> 2500	1180 (47.0)
Missing	76 (3.0)

### Missing values

The number of missing values of the CPG is presented in Table 2.

**Table 2:** Prevalence rates of chronic pain

**Criteria**	**N**	**% (95% CI)**	**Missing data**	**Valid cases**
Constant or frequently recurrent pain since > 3 months	710	28.3 (26.6- 30.2)	6	2502
Non-malignant pain	673	26.9 (25.2-28.6)		
Malignant pain	37	1.4 (1.35-1.45)		
SF-8 bodily pain intensity last 4 weeks (malignant pain excluded)			7	2464
None	1802	73.1 (71.3-74.9)		
Very slight	27	1.0 (0.9-1.1)		
Slight	106	4.2 (3.4-5.0)		
Moderate	371	15.0 (13.6-16.4)		
Strong	141	5.7 (4.6-6.6)		
Very strong	17	0.1 (0.08-0.12)		
SF-8 bodily pain very slight to very severe intensity last 4 weeks (malignant pain excluded) duration			6	663
3-6 months	88	13.2 (10.2-15.8)		
7-12 months	64	9.7 (7.5-11.9)		
1-3 years	181	27.3 (23.9-30.7)		
3-5 years	145	21.9 (18.8-25.0)		
5-10 years	101	15.2 (12.5-17.9)		
> 10 years	84	12.7 (10.2-14.2)		
Chronic pain stages (referred to total sample)			2	2452
I	271	11.1 (9.8-12.2)		
II	207	8.4 (7.3-9.5)		
III	122	5.0 (4.1-5.8)		
IV	60	2.4 (1.9-2.9)		
Chronic pain stages (referred to persons with chronic non-malignant pain)			2	660
I	271	41.1 (37.4-44.8)		
II	207	31.4 (27.9-34.9)		
III	122	18.5 (15.5-21.5)		
IV	60	9.0 (7.9-11.2)		

0.3% of the items of the SCQ and 0.2% of the items of the health care utilization questionnaire were not answered by the participants.

### Prevalence of chronic pain and of chronic pain stages

28.4% of the respondents reported constant or frequently recurring pain in the last 3 months. 5.3% of these persons reported malignant pain. These persons were excluded from further analyses. 26.9% reported pain of any severity in the SF-8 not associated with malignancies during the last 4 weeks and thus met the predefined criterion of chronic non-malignant pain. 21.5% met the SF-8 criterion of persistent bodily pain (at least moderate pain intensity in last 4 weeks). 19.5% of respondents met the criteria of chronic- non disabling pain and 7.4% met the criteria of chronic disabling pain (see Tables 2 and 3).

**Table 3:** Societal burden of chronic pain stages without and with adjustment for age and disease load (N = 2452)

**Pain stages**	**Variable**	**Mean (Minimum- maximum)**	**Unadjusted rate ratio (95% CI)**	**Adjusted rate ratio (95% CI)**
No pain	Days of sick leave/disability	3.9 (0–365)	1.0	1.0
Non-disabling pain		6.4 (0–336)	1.6 (1.5-1.7)	1.8 (1.7-1.9)
Disabling pain		24.8 (0–365)	6.4 (6.1-6.6)	6.8 (6.5-7.1)
No pain	Doctor visits	3.4 (0–50)	1.0	1.0
Non-disabling pain		8.4 (0–60)	2.5 (2.4-2.8)	1.7 (1.6-1.8)
Disabling pain		15.1 (0–70)	4.5 (4.3-4.7)	2.6 (2.4-2.7)
No pain	Nights in hospital	0.6 (0–80)	1.0	1.0
Non-disabling pain		1.7 (0–72)	2.7 (2.4-2.9)	1.5 (1.3-1.6)
Disabling pain		7.4 (0–110)	11.7 (10.8-12.7)	5.0 (4.5-5.5)

### Societal burden of chronic pain stages

Compared with no chronic pain, the rate ratio of days with sick leave/disability was increased 1.6 times for non-disabling pain and 6.4 times for disabling pain. After adjusting for age and disease load, the RRs was increased to 1.8 and 6.8 (see Table 3).

Compared with no chronic pain, the rate ratio of doctor visits was increased 2.5 times for non-disabling pain and 4.5 times for disabling pain. After adjusting for age and disease load, the RRs fell to 1.7 and 2.6 (see Table 3).

Compared with no chronic pain, the rate ratio of days in hospital was increased 2.7 times for non-disabling pain and 11.7 times for disabling pain. After adjusting for age and disease load, the RRs fell to 1.5 and 4.0 (see Table 3).

Lower education level (OR 3.27, 95% CI 1.70 to 6.29), disabling chronic pain (OR 3.30, 95% CI 1.76 to 6.21) and disease load with drug treatment (OR 1.70, 95% CI 1.41 to 2.05) predicted unemployment compared with persons working full or half time (see Table 4).

**Table 4:** Demographic and clinical predictors of unemployment

	**Working full-or >15 h/week (Referent)**	**Unemployed**	**Crude OR (95% CI) p-value**	Adjusted OR* (95% CI) p-value
**Gender** N (%)				
Female	608 (48.3)	64 (45.1)	Referent	0.89 (0.62-1.29) 0.54
Male	651 (51.7)	78 (54.9)	1.14 (0.80-1.61) 0.47	Referent
**Age** (years) Mean (SD)	43.44 (11.60)	43.44 (12.35)	1.00 (0.99-1.02) 0.99	0.98 (0.97-1.00) 0.94
**Education** N (%)				
High school or higher	282 (22.5)	11 (7.7)	Referent	Referent
Primary or secondary school	974 (77.5)	161 (92.3)	**3.45 (1.84-6.47) <0.0001**	**3.27 (1.70-6.29) <0.0001**
**Pain grade** N (%)				
No pain	1024 (82.2)	94 (68.6)	Referent	Referent
Non disabling pain	179 (14.4)	21 (15.3)	1.28 (0.78-2.11) 0.34	0.87 (0.5-1.5) 0.60
Disabling pain	43 (3.5)	22 (16.1)	**5.57 (3.20-9.71) <0.0001**	**3.30 (1.76-6.21) <0.0001**
**Disease load** (Number of diseases with drug treatment) Mean (SD)	0.37 (0.77)	1.06 (1.44)	**1.80 (1.54-2.09) <0.0001**	**1.70 (1.41-2.05) <0.0001**

*Adjusted for all other presented variables; Significant Odds ratios (OR) are marked as bold.

## Discussion

### Comparison with other studies

#### Prevalence of chronic pain

The prevalence rate of malignant and non-malignant pain was 26.0% in Kansas [16] and was 28.3% in Germany. 26.8% of adult Danes reported that they have chronic pain defined by the question “Do you have chronic/long-lasting pain lasting 6 months or more?” [25]. In contrast, the prevalence of chronic pain might be higher in Asia and lower in New Zealand. The prevalence of chronic pain (defined by positive replies to both question “Are you currently troubled by physical pain or discomfort, either all the time, or on and off?”; and “Have you had this pain or discomfort for more than 3 months?”) in adults in Hong Kong was 34.9% [26]. The prevalence of chronic pain (defined by a positive response to the question “did you experience pain that is present almost every day, but the intensity of the pain may vary; pain that has lasted or is expected to last six months or more”) in persons aged 15 years and over in New Zealand was 16.9% [10].

The comparison with the findings of other studies using other definitions of chronic pain highlights the impact of wording on the prevalence rates of chronic pain in the general population. The prevalence rates ranged from 18.5% to 19.6% in three Canadian surveys with persons aged 20 years plus conducted in 1994, 1996 and 2008. Chronic pain was assessed by a “no” response to the question “Are you usually free of pain or discomfort?” [27]. In contrast, the prevalence rate of chronic pain was 32.9% in the German 2012 population survey [14] by asking for pain over the past seven days in 19 pain areas of the body in the regional pain scale [28] and in case of pain reports in the past seven days by a positive response to the question “Were these symptoms above generally present for at least 3 months?”

Recently, the bodily pain scale of the Short-From Health Survey 8 has been recommended as standard measure of persistent bodily pain which is not specific to a single anatomical site [5]. When defined as moderate pain or more on at least 3 of 4 consecutive measurements, the prevalence of persistent bodily pain was 26% in the general population of Norway [6]. Using the 4-weeks time interval of the German SF-8, the prevalence of moderate pain or more was 21.5% in our study. In a survey including 16 European countries and Israel, 17% of German and 30% of Norwegian participants aged 18 years or more reported moderate or severe pain of at least six months duration [11]. Thus, the prevalence rates of moderate and severe pain showed remarkable differences between European countries.

#### Prevalence of chronic pain grades

The prevalence of chronic pain based on the IASP-definition was 35.5% in Irish adults identified by a general practitioner data base [29]. Using the CPG, the prevalence rate of disabling pain was 37.0% of persons with chronic pain [29]. 21.5% of the persons with chronic pain in the Hong Kong survey [26] met the criteria of disabling pain. The percentage of persons with disabling pain in the chronic pain group in our sample was 27.6%. The differences in the prevalence rates of chronic pain and its grades might be explained by differences of setting and culture. As with chronic pain, the prevalence rates of disabling pain depend on the instruments used. In the German 2012 survey of our study group [14], 7.7% of the general population met the criterion of disabling pain, as defined by the regional pain scale [28] and by a quality of life questionnaire of the European Organisation for Research and Treatment of Cancer QLQ-C30 Version 3.0 [30]. In the Canadian surveys the percentages of persons with disabling pain defined by any limitation of activities by pain or discomfort ranged from 11.4% to 13.3% [27].

#### Societal burden

In accordance with the New Zealand study [10], accumulated disease load was independently associated with chronic pain in our sample. In line with an UK prospective population with persons aged 50 years plus [31], those with interfering pain reported more comorbidities in our sample. Healthcare resource utilization and periods of disability increased with chronic pain stage in our sample as it did in persons reporting severe pain compared to those with slight or moderate pain in the 2008 National Health and Wellness Survey conducted in five European countries [32]. Most notably, not only stages of chronic pain, but also disease load increased days with disability/sick leave, doctor visits and nights in hospital as well as unemployment in our sample. In addition, low educational level predicted unemployment as did in a US survey [33]. Based on the biopsychosocial model of chronic pain we assume that somatic diseases and mental disorders predispose to chronic pain [34]. Societal inequalities increase the risk of somatic diseases and mental disorders and vice versa [35].

### Limitations

a). The response rate of 58% was comparable with the one of recent surveys conducted in Europe [14,25], USA [16] and Asia [26]. However, 42% of persons addressed were non-responders. We do not know if there were relevant differences between the participants of the survey and the ones who did not participate. Data protection laws in Germany did not allow the assessment of demographic characteristics of non-respondents. Marginal differences in demographic characteristics between this sample and the population imply that true rates may vary slightly from those we report. b). We did not formally use the IASP definition of chronic pain [15] to identify persons with chronic pain. c). The design of the study precluded an independent medical assessment for somatic diseases and mental disorders. d). Self-reports of days of sick leave and health care use over a 12 months period are subject to memory bias. e). Due to German laws of data protection we could not access health care use and days of sick leave data of the participants available at health insurance companies. f). Finally, the cross-sectional design of the study did not allow determining time course or inferring causality between chronic pain and other variables. We asked about pain over the last three months and burden included those three months. Although we cannot attribute with certainty that the burden identified was entirely due to chronic pain, it is highly likely that at least a very large proportion of the increased burden should be attributed to chronic pain.

## Conclusion

Pain societies and researchers have made considerable efforts to promote the awareness of the public that chronic pain is a major health problem [2-4]. Our data confirm the societal impact associated with chronic pain. However, the societal burden is not only due to chronic pain, but also due to disease load and social inequalities. Pain, somatic diseases, mental disorders and social disparities cannot be disentangled. More longitudinal studies are necessary, to investigate predictors of different trajectories of chronic pain such as are persistent severe/disabling, persistent mild/non-disabling and fluctuating pain [36]. The identification of modifiable individual psychological features of persons at increased risk for persistent severe/disabling pain and of protective factors promoting mild and non-disabling pain could target the use of scarce resources [29]. A standardization of pain measurements in epidemiology research of chronic pain is necessary to increase the credibility of the findings of the pain research community [5]. The pain scale of the SF-8 has been suggested as standard measure of persistent bodily pain in cross-sectional surveys [5]. In addition, the chronic pain grade scale [9] enables a distinction between disabling and non-disabling pain. Measurement of chronic pain independent of comorbid conditions and adjustment for associated diseases by comorbidity indices are necessary for more accurate prevalence estimates of chronic pain [10].

## Abbreviations

CI: Confidence interval; CPG: Chronic Pain Grade Questionnaire; IASP: International Association of the Study of Pain; SCQ: Self-administered comorbidity questionnaire; SF: Short Form Health Survey.

## Competing interests

WH received a consulting honorarium by Daiichi Sankyo and honororia for educational lectures by Abbott and Pfizer within the last 3 years. PH received honoraria for educational lectures by Eli Lilly and Novartis within the last 3 years. The other authors have no conflicts of interest to declare.

## Authors’ contributions

All authors discussed the results and commented on the manuscript. WH provided clinical and scientific input in the design and analysis of study and in data interpretation; author of the manuscript. GS and FW were responsible for data analysis and reviewed the manuscript. PH provided clinical and scientific input in design and analysis of the study and in data interpretation. EB and AH were the main responsibles for the organization of the population survey, provided expertise on the methodology, provided scientific input in the data interpretation and reviewed the manuscript. All authors read and approved the final manuscript.

## Authors’ information

WH is the general secretary of the German Association of Interdisciplinary Pain Therapy, an umbrella organisation of medical and psychological societies.

## Pre-publication history

The pre-publication history for this paper can be accessed here:

http://www.biomedcentral.com/1471-2458/14/352/prepub

## Supplementary Material

Additional file 1 Comparison of demographic data of the study sample with the general German population. Click here for file
