# Supplementary material for: Untying chronic pain: prevalence and societal burden of chronic pain stages in the general population - a cross-sectional survey
Source: BMC Public Health. 2014 Apr 13;14:352. doi: 10.1186/1471-2458-14-352 (PMC4022433; doi:10.1186/1471-2458-14-352)
Supplement: Additional file 1 — Comparison of demographic data of the study sample with the general German population. [file 1471-2458-14-352-S1.doc]

Additional file 1: Comparison of demographic data of the study sample with the general German population (24)

|  | **Total German population 2011**  **N= 80 219 695**  **(%)** | **Study sample**  **2013**  **N= 2508**  **(%)** |
| --- | --- | --- |
| **Age**  ≤ 29 years  30-49 years  50-64 years  ≥ 65 years | 29.8 *  28.2  20.8  21.2 | 17.8 **  31.0  26.8  24.3 |
| **Women** | 51.3 | 53.2 |
| **German citizenship** | 92.3 | 96.2 |
| **Highest educational degree**  No school finished  In school  Primary or secondary school  finished  High school or higher  Missing data | 4.7  4.4  62.5  28.3 | 2.7  3.1  75.7  18.1  0.4 |
| **Employment**  Without job  Working  Not working | 2.7  50.2  47.1 | 5.7  53.1  47.2 |

* Persons aged 0-29 years; ** Persons aged 14-29 years
